# Oncological and reproductive outcomes of adenocarcinoma in situ of the cervix managed with the loop electrosurgical excision procedure

**DOI:** 10.1186/s12885-018-4386-6

**Published:** 2018-04-24

**Authors:** Huimin Bai, Jun Liu, Qiuxi Wang, Ying Feng, Tong Lou, Shuzhen Wang, Yue Wang, Mulan Jin, Zhenyu Zhang

**Affiliations:** 1 Department of Obstetrics and Gynecology, Beijing Chao-Yang Hospital, Capital Medical University, No. 8, North Road of Workers Stadium, Chaoyang District, Beijing, 100020 China; 2 Department of Pathology, Beijing Chao-Yang Hospital, Capital Medical University, Beijing, China

**Keywords:** Adenocarcinoma in situ, Loop electrosurgical excisional procedure, Oncological and reproductive outcomes

## Abstract

**Background:**

The standard treatment for cervical adenocarcinoma in situ (AIS) is hysterectomy, which is a more aggressive treatment than that used for squamous intraepithelial lesions. Several previous studies have primarily demonstrated that the loop electrosurgical excision procedure (LEEP) is as safe and effective as cold knife cone (CKC) biopsy when AIS is unexpectedly found in a loop excision. This study evaluated the safety of LEEP as the initial treatment for patients with AIS who were strictly selected and evaluated before and after loop resection.

**Methods:**

The oncological and reproductive outcomes of a series of AIS patients who underwent LEEP as the initial treatment between February 2006 and December 2016 were retrospectively evaluated.

**Results:**

A total of 44 women were eligible for analysis. The mean age at diagnosis was 36.1 years, and 14 patients were nulliparous. Multiple lesions were identified in 4 (9.1%) patients. Either hysterectomy (6 patients) or repeat cone biopsies (3 patients) were performed in 8 of the 10 patients who presented positive or not evaluable surgical resection margins (SMs) on the initial LEEP specimens. Residual disease was detected in two patients. All patients were closely followed for a mean of 36.9 months via human papillomavirus testing, PAP smears, colposcopy, and endocervical curettage when necessary. No recurrences were detected. Of the 16 patients who desired to become pregnant, 8 (50%) successfully conceived, and the full-term live birth rate was 83.3% among this subgroup.

**Conclusions:**

LEEP with negative SMs was a safe and feasible fertility-sparing surgical procedure for patients with AIS, and the obstetric outcome was satisfactory. However, long-term follow-up is mandatory.

## Background

Adenocarcinoma in situ (AIS) of the cervix is a rare condition and is considered a precursor of invasive adenocarcinoma. The standard treatment for cervical AIS is hysterectomy, which is a more aggressive treatment than that used for squamous intraepithelial lesions. Acceptance of the standard hysterectomy approach may be due to the distinct biological behaviors of adenocarcinomas, such as their multifocality, the localization of lesions high up in the endocervical canal, and their association with occult invasive adenocarcinoma [1, 2]. However, several previous studies have suggested that AIS, similar to its squamous cell carcinoma (SCC) counterpart, is located around the transformation zone of the cervix, and most tumors are unifocal [3, 4]. Due to improved detection methods, the increasing incidence of human papillomavirus (HPV) 18 infection, and the increasing use of oral contraceptives, the incidence of AIS has increased over the past 30 years, especially in young women [5, 6]. The current trend to delay childbearing among women has also led to an increase in requests for fertility-sparing procedures, such as cold knife cone (CKC) biopsy or loop electrosurgical excision procedure (LEEP), to manage such lesions.

Conization, especially CKC biopsy, has been used for the diagnosis and treatment of cervical intraepithelial neoplasia (CIN), including carcinoma in situ. However, the recently introduced LEEP has become more common due to its technical ease and lower morbidity [7, 8]. In addition, LEEP is usually performed under regional anesthesia in an outpatient clinic with significantly lower costs. However, the treatment role of LEEP for managing AIS remains controversial. LEEP has been associated with significantly higher rates of positive section margins [9–11] and a higher recurrence risk than CKC biopsy [12]. In contrast, recent retrospective studies have demonstrated that LEEP is as safe and effective as CKC biopsy when AIS is unexpectedly found in a loop excision [6, 13, 14].

In addition to CKC biopsy and hysterectomy, LEEP has been recommended as an option for managing patients with AIS under fully informed consent and strict surveillance in our institute for the past ten years. The oncological and subsequent reproductive outcomes of these patients are reported in this study, with a specific focus on evaluating the safety and feasibility of this treatment method.

## Methods

Patient records and information were anonymized and de-identified prior to analysis, and therefore, consent was not necessary. The study protocol was approved by the ethics committee of Beijing Chao-Yang Hospital, Affiliated China Capital Medical University. The study was carried out in accordance with the ethical standards of the responsible committee on human experimentation (institutional and national) and with the Helsinki Declaration of 1975, as revised in 2008. All consecutive patients between February 2006 and December 2016 who were managed with LEEP as the initial therapy were registered within a computerized cancer database at Beijing Chao-Yang Hospital. Patients suitable for LEEP management had to fulfill the following inclusion criteria: age ≤ 45 years, histologically proven AIS, a strong desire to maintain fertility, and be available for strict follow-up. Patients with coexisting invasive adenocarcinoma or SCC on either the initial cervical biopsy or on the initial LEEP specimen were excluded. Patient information, including demographic characteristics, Papanicolaou smear results, colposcopic findings, the LEEP method, pathology results, disease status at the last contact, and postoperative reproductive outcomes, was collected and evaluated.

A colposcopic evaluation was performed using a Zeiss OPM1F colposcope (Karl Zeiss, Jena, Germany). During the examination, both acetic acid and Lugol’s iodine solution were used. Based on the visibility of the squamous-columnar junction (SCJ) during the colposcopy, this region was classified as type I (visible), II (the lower boundary was visible, but the upper boundary was not visible), or III (not visible). Directed punch biopsies were taken to examine the suspicious sites.

LEEP was performed in patients with a glandular lesion who desired non-radical treatment or the preservation of fertility after being fully informed of the risk of persistent or recurrent disease. This conservative approach was completed in the clinic under local anesthesia. During the conization procedure, the cervix was swabbed with Lugol’s iodine solution before resection. This technique is useful for locating the ectocervical margins of the lesion, which has been described previously [15]. When the margin status was ill-defined, additional sections were obtained. The 12 o’clock position of the LEEP specimen was sutured for orientation, and the inner surface was inked. The specimens were fixed in 10% neutral formalin solution. The cone margins were marked with indelible ink prior to serial sectioning. The conization specimens were then submitted in entirety for microscopic evaluation, and sections stained with hematoxylin and eosin were used for pathology examination.

The histological and morphological criteria for AIS included cervical glandular pseudostratified epithelial cells, enlarged and hyperchromatic nuclei, and mitotic figures without stromal invasion. The marginal status was interpreted as positive when AIS or neoplastic epithelium of any grade was identified in any of the ectocervical, endocervical or deep margins [16]. The presence of normal cervical epithelium had to be detected at the margin in all sections containing neoplastic epithelium to qualify as a negative margin of conization; in addition, the distance between the neoplastic cells and the margin had to be no less than 1 mm for the margin status to be defined as negative [17]. The margin status was interpreted as not evaluable if any margin lacked surface epithelial cells. Two independent pathologists with extensive experience specifically in gynecologic pathology reviewed all the cytological and histological slides for the purposes of this study. These reviewers were blinded to the patients’ outcomes. Disagreements between the two investigators were resolved by discussion.

After completing the treatment, the women were strictly followed-up monthly for the first half-year, every 3 months for the second half of the year, and every 6 months thereafter. The results from liquid-based Papanicolaou smears, HPV tests, and colposcopies were examined every 3–6 months. The Hybrid Capture II (HCII) assay was used for HPV testing, and the specimens were tested with probe B. Colposcopy was also routinely performed considering the potentially aggressive nature of adenocarcinoma. An endocervical curettage (ECC) was administered when the examination of cervical transformation area was unsatisfactory. AIS or invasive cancer was regarded as residual or recurrent disease if found in the specimen obtained during a subsequent procedure within or more than 3 months, respectively, after the initial LEEP. Efforts were made to contact patients by telephone or letter to obtain this information when regular follow-up information was not available.

## Results

During the study period, a total of 45 patients with cervical AIS who underwent LEEP as the initial therapy met the inclusion criteria of this study. One patient with concurrent SCC on the initial LEEP specimen was excluded from further analysis. The clinicopathological characteristics of the 44 included eligible patients are shown in Table 1. The mean age at the time of diagnosis was 36.1 (range: 28–55) years. Fourteen patients (31.8%) were nulliparous. No patients in our series were using an oral contraceptive. A few patients presented with certain symptoms, such as contact bleeding (2 cases) and vaginal discharge (1 case), but most were asymptomatic (41 cases, 93.2%). These women were referred for colposcopy due to an abnormal PAP smear (5 cases), positive high-risk HPV testing (5 cases), or both (34 cases). The upper boundary of the SCJ was not visible in 29 patients (65.9%), leading to an unsatisfactory colposcopic examination.

**Table 1:** Clinopathological characteristics of the 44 patients with AIS of the cervix who were treated with LEEP

Parameters	Number of patients	Percent (%)
Age at diagnosis, (Mean;range)	36.1 ± 14.2 (28–55)	
Baseline parity		
Nulliparous	14	31.8
Pluriparous	30	68.2
Presentation		
Contact bleeding	2	4.5
Vaginal discharge	1	2.3
Asymptomatic	41	93.2
Referral Cytology		
ASCUS	13	29.5
AGC	4	9.1
LSIL	8	18.2
HSIL	13	29.5
AIS	1	2.3
NILM	5	11.4
High-risk HPV		
Negative	4	9.1
Positive	39	88.6
Unkonwn	1	2.3
Height of LEEP specimens(cm; range)	1.1 ± 0.2 (0.7–2)	
Depth of LEEP specimens(cm; range)	1.5 ± 0.3 (1–2)	
Status of LEEP margin		
Negative	33	70.5
Positive	10	29.5
Not evaluable	1	2.3
AIS detected by		
Papanicolaou test	1	2.3
Punch biopsy	12	27.3
LEEP only	31	70.5
Multifocality		
Yes	4	9.1
No	40	90.9
Coexisting squamous disease		
CIN I	5	11.4
CIN II	8	18.2
CIN III	25	54.5
Absent	6	13.6
Follow-up (month;range)	36.9 ± 18.7 (3–101)	
Status at the last contact		
Abnormal cytology	1	2.3
Positive high-risk HPV	6	13.6
Both positive	1	2.3
Both negative	36	81.8

Note:*AIS* adenocarcinoma in situ, *LEEP* loop electrosurgical excisional procedure, *ASCUS* atypicai squamous cells, AGC,atypical glandular cell, *HSIL* high-grade squamous intraepithelial lesions, *NILM* negative for intraepithelial lesion or malignancy, *CIN* cervical intraepithelial lesion

The resection margins of the LEEP specimens were negative in 33 (31.5%) patients, positive in 10 (29.5%) patients, and not evaluable in one (2.3%) patient. Four (9.1%) patients presented multifocal lesions. Coexisting squamous lesions were identified in 38 (86.4%) patients. Of the 11 patients who had positive or not evaluable resection margins on the LEEP specimens, 9 underwent subsequent procedures, including hysterectomy (6 cases), CKC (2 cases) or a second LEEP (1 case). Clear margins were obtained on the specimens of subsequent procedures, and residual disease was identified in 2 patients: AIS in 1 case and CIN3 in 1 case. The remaining 2 patients refused any further surgical treatment.

All 44 patients were closely followed up for a mean of 36.9 months. During the follow-up period, 7 patients received further histological evaluation through punch biopsies (2 cases), ECC (2 cases), and LEEP (5 cases) due to an abnormal PAP smear and/or positive high-risk HPV testing in combination with an abnormality detected during colposcopy. Of these patients, 6 had low-grade abnormalities, and one had negative findings. No recurrent disease was identified in the specimens from these procedures. No further procedures were performed on these patients. At the last contact, 7 patients still had an abnormal PAP smear (1 case), positive high-risk HPV testing (5 cases) or both (1 case). No patients died of the disease, and no patients had suspicious lesions detected through gynecological examination and colposcopy.

In this series, 8 (50%) of the 16 patients who desired to become pregnant conceived naturally (7 cases) or through assisted reproductive technology (1 case). One of these 8 patients suffered from spontaneous abortion at 12 weeks of gestation due to incompetence of the cervix. At the last contact, two patients were still pregnant at week 11 and week 16 of gestation without any abnormal clinical manifestations. The remaining 5 (83.3%) women had full-term pregnancies and gave birth to live newborns through Cesarean section (3 cases) and vaginal (2 cases) delivery.

## Discussion

LEEP with negative SMs is regarded as sufficient treatment for SCC in situ of the cervix. Traditionally, cervical AIS has been treated more aggressively than squamous intraepithelial lesions because this tumor type has been thought to be multifocal and located high in the cervical canal [1, 2]. However, more recent studies have demonstrated that most cases of AIS are unifocal and originate at the SCJ or within the transformation zone [3, 4]. The tumor generally extends proximally in a contiguous manner into the endocervical canal, mostly within 3 mm of the SCJ [18, 19]. A multifocal distribution of AIS has been found in 6.3% to 14.3% of cases [3, 20]. In the present study, multifocal lesions were present in 4 of 44 patients (9.1%), which falls between the previously reported rates of multifocal lesions. These results suggest that the probability of multifocal disease in AIS is low but not negligible.

The risk of residual disease associated with conization when managing patients with AIS who desire to preserve their fertility is a main concern of gynecologists. For a definitive histological diagnosis of AIS, the whole cervical transformation zone must be considered to ensure the exclusion of potential concurrent invasive adenocarcinoma [21]. CKC has been a more commonly recommended therapeutic method than LEEP, mainly because CKC can provide a cone specimen with a relatively greater depth and larger volume [22]. Three previous systematic reviews [23–25] and a meta-analysis [14] demonstrated that LEEP had a significantly higher rate of positive margins than CKC. A positive SM is an effective predictor of residual disease [16, 17]. However, in exploring the details of the data in these studies, there was a notable decreasing trend in the rate of positive margins with LEEP over time, with rates of 51% in 2014 [24] and 44% in 2017 [14]. This parameter for LEEP was only 29.5% in this study, which is comparable to that of CKC reported in the literature (30% and 29% by Baalbergen et al. and Jiang et al., respectively) [14, 24]. These results seem toimply a tendency of employing LEEP as the preferred treatment for AIS. For patients with positive SMs, hysterectomy or repeat cone biopsies (CKC or LEEP) are recommended.

Possibly due to the multifocality of adenocarcinoma, a negative conization margin in AIS is reliably predictive of a low but not nonexistent incidence of residual glandular neoplasia [16, 26]. Tierney et al. [27] combined the results from 18 studies published in the literature and found that 18% of patients with negative margins still had residual AIS, and 2% had cancer. An absence of neoplastic epithelium at all cone margins has been used as the general criterion for a negative conization margin [16, 19]. In this analysis, a distance of neoplastic cells from the margin no shorter than 1 mm was also considered when determining the margin status. According to this standard, the reported risk of residual disease has been reported to be as low as 7.7% (1/13) and zero (0/26) [11, 17]. Thus, the use of strict evaluation criteria for a negative margin of conization could effectively reduce the risk of residual disease.

The use of ECC in combination with cone biopsy for cervical AIS could also provide valuable predictive information regarding residual AIS [27, 28]. If the post-conization ECC is positive, regardless of the margin status, the risk of residual glandular neoplasia is very high (100%) [29]. In another study, 92% of patients in whom both conization SMs and ECC were positive for the presence of AIS had residual AIS or invasive adenocarcinoma [27]. In contrast, these two studies reported that women with AIS who had both a negative conization margin and a negative ECC had an 11.1% and 14% risk of residual disease. In our opinion, this procedure is specifically recommended when the examination of cervical transformation area is unsatisfactory. This procedure may be helpful in detecting residual disease after initial LEEP treatment and during the follow-up period.

Compiled retrospective data have demonstrated that LEEP with negative SMs is equally safe and effective in managing patients with AIS who desire to preserve their fertility [6, 13, 14]. In addition, LEEP is technically easier and less expensive and is associated with less morbidity, such as pain, cervical stenosis, and hemorrhage, than CKC [7, 8]. More importantly, LEEP has clinical advantages over CKC in terms of future pregnancies. Two large meta-analyses showed that patients treated with CKC had a significantly increased risk of preterm delivery compared with those treated with LEEP [30, 31]. In addition, Noehr and his colleagues [32, 33] demonstrated in a large cohort study that an increased depth of the loop cone is directly associated with an increased risk of preterm delivery, with a 6% increase in risk for each additional millimeter of tissue excised. The rate of positive margins for LEEP with a mean cone depth of 8 mm was not significantly higher than that for CKC with a mean cone depth of 15 mm. In this study, the mean cone depth of LEEP was 11 mm, and the full-term live birth rate was 83.3% (5/6). However, due to the very small sample size of these studies, further clinical trials are warranted to explore the optimal cone depth for LEEP to balance the risk of positive margins and satisfactory obstetric outcomes.

In this analysis, no patients developed recurrence during the study period. Our preliminary data suggested that LEEP with negative SMs was feasible for use as the sole treatment for patients with AIS. However, the follow-up period was relatively short (approximately 3 years). The reported recurrence rate after achieving negative margins on cone specimens in previous studies ranged widely from 1.1% to 46.7%, and the calculated weighted average was 6.3% (42/669) [20]. Possible explanations for this phenomenon included inconsistent pathology interpretation, persistent HPV infections, and/or multifocal disease [9, 34]. Consequently, achieving negative resection margins on a LEEP specimen cannot completely preclude the possibility of recurrent AIS. Long-term surveillance and education regarding the risks of recurrent disease is required. Data regarding the long-term follow-up strategy for patients with AIS undergoing uterus-sparing procedures have rarely been reported in the literature. Furthermore, the sensitivity of either cervical cytology or colposcopy alone in predicting AIS is suboptimal. AIS cases have been detected in only 10% to 30% of cases through Papanicolaou testing and 30% to 50% of cases through colposcopically directed biopsy prior to cervical conization [35–37]. In this analysis, 2.3% and 27.3% of AIS patients were diagnosed through Papanicolaou testing and colposcopically directed biopsy prior to cervical conization, respectively. The incidence of coexisting squamous intraepithelial lesions was 84.4% in our series, which was higher than that in previous reports ranging from 28% to 64% [1, 37, 38]. The presence of coexisting squamous intraepithelial lesions may be related to the low sensitivity of the cervical cytology, which may obscure the abnormality of the glandular component. Similar to CIN, AIS is closely related to high-risk HPV infection, predominantly HPV 18, with an incidence ranging from 25 to 88% [16, 39, 40]. In this analysis, the follow-up strategies for patients with AIS who received LEEP as the initial treatment included repeated evaluation using cervical cytology, HPV DNA testing, and colposcopy with ECC, which is recommended in the American Society for Colposcopy and Cervical Pathology 2006 consensus guidelines. Considering the aggressive nature of adenocarcinoma, hysterectomy may always be indicated during surveillance.

Due to its rarity and potentially aggressive nature, most cases of AIS in the literature were generally found unexpectedly during a loop excision procedure, leading to the presentation of retrospective results. In this study, we compiled a series of consecutive patients with AIS who received LEEP as the sole treatment. All the patients included in this study were strictly selected and evaluated before and after loop resection. Despite the limitations of this study, such as its single-institute design, small sample size, and relatively short follow-up period, our data provide objective and convincing insights into the management of this disease.

## Conclusions

Strict evaluation criteria for a negative margin of conization combined with ECC effectively reduced the risk of residual disease. LEEP with negative SMs was a safe and feasible fertility-sparing surgical procedure for patients with AIS, and the obstetric outcome was satisfactory. Nevertheless, further clinical trials are warranted to validate these results.

## Abbreviations

AIS: Adenocarcinoma in situ
CIN: Cervical intraepithelial neoplasia
CKC: Cold knife cone
ECC: Endocervical curettage
HCII: Hybrid Capture II
HPV: Human papillomavirus
LEEP: Loop electrosurgical excision procedure
SCC: Squamous cell carcinoma
SCJ: Squamous-columnar junction
